# Cannabinoid Hyperemesis Syndrome Presenting as Postoperative Nausea and Vomiting in a Chronic Cannabis User: A Case Report

**DOI:** 10.7759/cureus.87990

**Published:** 2025-07-15

**Authors:** Justin B Atkins, Daniel Levine, Laura Shaw

**Affiliations:** 1 Kirk Kerkorian School of Medicine, University of Nevada, Las Vegas (UNLV), Las Vegas, USA; 2 Family Medicine, Kirk Kerkorian School of Medicine, University of Nevada, Las Vegas (UNLV), Las Vegas, USA

**Keywords:** abdominal pain, anesthesiology implications, antiemetic resistance, cannabinoid hyperemesis syndrome, cannabis withdrawal, chronic cannabis use, cyclic vomiting, hot water bathing, perioperative complications, postoperative nausea

## Abstract

Cannabinoid hyperemesis syndrome (CHS) is a paradoxical condition seen in chronic cannabis users, marked by recurrent nausea, vomiting, and abdominal discomfort. Although more widely recognized in emergency medicine, CHS remains underdiagnosed in the perioperative setting, where its symptoms may be misattributed to common postoperative phenomena such as anesthetic effects, opioid-induced nausea, or surgical complications. This diagnostic gap can delay appropriate management and lead to unnecessary interventions.

We report the case of a 40-year-old woman with a two-year history of daily cannabis use who underwent a laparoscopic hysterectomy, mid-urethral sling placement, and pelvic organ prolapse repair. In the immediate postoperative period, she experienced persistent nausea and vomiting despite the administration of multiple antiemetics, including ondansetron and metoclopramide, and opioids for pain control. By postoperative day 2, vomiting occurred in discrete, refractory episodes despite continued pharmacologic management, prompting concern for an atypical cause. The patient was placed on nil per os (NPO) status, but symptoms escalated on postoperative day 3, culminating in a prolonged episode of emesis accompanied by hematemesis and hallucinations.

At this stage, CHS was strongly suspected given her chronic cannabis use, clinical trajectory, and lack of response to standard therapies. Supportive care was intensified with intravenous hydration and electrolyte replacement for significant hypokalemia and hypophosphatemia. The patient’s condition stabilized over the next 24 hours, with gradual resolution of symptoms and resumption of oral intake by postoperative day 4. She was discharged in stable condition with a tailored regimen of antiemetics, analgesics, and counseling on cannabis cessation. She abstained from cannabis throughout hospitalization.

This case highlights a critical but underrecognized cause of refractory postoperative nausea and vomiting (PONV). In patients with a history of chronic cannabis use, perioperative teams should maintain a high index of suspicion for CHS when standard antiemetic regimens fail. Early identification not only prevents unnecessary diagnostic testing and extended hospitalization but also enables more effective patient education and targeted counseling. Broader awareness of CHS among surgical and anesthesia teams can improve outcomes through timely diagnosis, supportive care, appropriate discharge planning, and public health efforts to raise awareness of cannabis-related complications.

## Introduction

Cannabinoid hyperemesis syndrome (CHS) is a clinical condition associated with chronic cannabis use, marked by cyclical episodes of nausea, vomiting, and abdominal pain [1]. As cannabis legalization expands, CHS is increasingly seen in emergency departments, yet remains underrecognized in perioperative settings [2]. Patients with CHS often report temporary relief of symptoms through compulsive hot bathing, a unique behavioral hallmark that can aid in diagnosis [3].

The paradox of cannabis-induced hyperemesis is often masked by its known antiemetic properties, leading both patients and healthcare providers to misattribute symptoms to more common causes such as gastroenteritis, adverse drug reactions, or postoperative complications [4]. In surgical populations, this diagnostic uncertainty can lead to delayed recovery, unnecessary imaging or laboratory workups, and ineffective antiemetic regimens. Awareness of CHS remains limited among anesthesiologists and surgeons, despite the rising prevalence of cannabis use across diverse patient populations [5]. Cannabis use may also influence perioperative care by altering anesthetic requirements and opioid tolerance, making its preoperative disclosure and recognition even more clinically significant.

This case underscores the importance of identifying CHS in the postoperative setting, particularly when standard antiemetic therapies fail and no clear surgical complication is evident. It also highlights the value of routine preoperative screening for chronic cannabis use as part of the anesthetic risk assessment and calls for enhanced education among perioperative clinicians to reduce diagnostic delays and unnecessary interventions and optimize patient outcomes.

## Case presentation

A 40-year-old woman with a two-year history of daily cannabis use presented for elective laparoscopic surgery, including vaginal hysterectomy, mid-urethral sling placement, and pelvic organ prolapse repair. The procedures were completed successfully without intraoperative complications, lasting approximately three hours under general anesthesia.

On day 0, the patient developed nausea and vomiting in the immediate postoperative period. Initial management included ondansetron 4 mg IV every 8 hours as needed for nausea and analgesia with morphine 4 mg IV every 4 hours and meperidine 25 mg IV every 6 hours as needed for pain. At this stage, her symptoms were attributed to routine postoperative nausea and vomiting (PONV), which is common following pelvic surgery and opioid use.

By postoperative day 1, the patient’s nausea and vomiting persisted despite receiving ondansetron 4 mg IV every 8 hours and initiation of metoclopramide 10 mg IV every 8 hours as needed. The ongoing emesis prompted consideration of atypical causes beyond routine PONV. Differential diagnoses included opioid-induced ileus, anesthetic sensitivity, surgical complications such as bowel injury, or medication side effects. Physical examination of the abdomen revealed a soft, nondistended abdomen with normoactive bowel sounds and no tenderness. Given the stable clinical status and reassuring abdominal exam, no imaging studies were performed at this time.

On postoperative day 2, the patient experienced three discrete episodes of vomiting despite continued administration of ondansetron 4 mg IV every 8 hours and metoclopramide 10 mg IV every 8 hours. Pain was managed with oxycodone 5 mg orally every 6 hours as needed, with no increase in opioid dosing compared to day 1. At this juncture, a detailed substance use history was revisited, during which the patient disclosed chronic daily cannabis use for approximately two years, a fact not initially volunteered. She reported inhaling approximately 1 g of cannabis flower per day, purchased from a licensed dispensary, with an estimated tetrahydrocannabinol (THC) concentration of 18%-22%. Given the persistent, refractory nature of her symptoms and absence of surgical complications, the patient was placed on nil per os (NPO) status to limit gastrointestinal stimulation. CHS was suspected based on the clinical picture. Notably, the patient reported frequent use of hot showers prior to hospitalization as a means of symptom relief, a hallmark behavioral clue supporting the diagnosis. Laboratory evaluation revealed mild hypokalemia (3.2 mmol/L) and hypophosphatemia (2.0 mg/dL), which were addressed with intravenous electrolyte supplementation. Imaging was not pursued due to stable vital signs and benign abdominal examination.

On postoperative day 3, the patient experienced a prolonged vomiting episode that began around 7 PM the previous evening and continued intermittently until approximately 5 PM the following day. This episode was notable for associated hematemesis and visual hallucinations lasting several hours. During this period, antiemetic therapy with ondansetron and metoclopramide was maintained, and pain control involved a stable regimen of oxycodone 5 mg orally every 6 hours as needed; no escalation in opioid dosage was required. Laboratory tests revealed worsening hypophosphatemia (1.8 mg/dL) and hypokalemia (2.9 mmol/L), which were promptly corrected with intravenous electrolyte replacement and approximately 2 L of isotonic saline over 24 hours. Despite these interventions, vomiting persisted until supportive care efforts began to take effect and symptoms gradually resolved. The patient’s combination of gastrointestinal symptoms, neuropsychiatric manifestations, chronic cannabis use, and lack of response to conventional antiemetics reinforced the diagnosis of CHS. Differential diagnoses such as intra-abdominal pathology and drug-induced toxicity were excluded through stable vital signs, unremarkable abdominal exams, and absence of imaging abnormalities. This confirmed the diagnosis based on the classical triad of chronic cannabis use, cyclic vomiting, and symptom resolution with supportive care.

**Figure 1:**
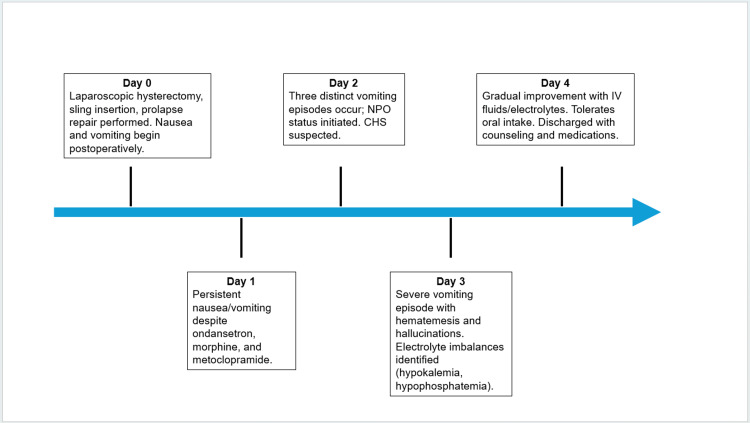
Clinical course of the patient with cannabinoid hyperemesis syndrome (CHS) following elective pelvic surgery, illustrating postoperative symptom progression and resolution timeline.

On postoperative day 4, the patient’s symptoms steadily improved. She was able to tolerate oral intake, starting with ice chips and advancing to soft solids, including grapes. After remaining free of nausea and vomiting for over 12 hours, she was deemed stable for discharge. Her final electrolyte panel was within normal limits, and all medications had been well tolerated. She was discharged with oxycodone-acetaminophen (5-325 mg every 6 hours as needed) for pain, ondansetron (4 mg every 8 hours as needed) for nausea, methocarbamol (500 mg every 8 hours as needed) for postoperative muscle spasms, and oxybutynin (5 mg daily) for urinary urgency related to her mid-urethral sling. She received extensive counseling on CHS and was advised to discontinue cannabis use. The patient’s complete postoperative course and corresponding interventions are summarized in Figure 1.

## Discussion

CHS remains a diagnostic challenge in postoperative patients due to its symptom overlap with more common conditions such as PONV, ileus, and medication side effects. While PONV typically responds to antiemetics, CHS is characteristically refractory to these treatments and often persists despite standard interventions [6]. This distinction is critical but frequently overlooked in perioperative settings, where nausea and vomiting are often attributed to anesthetic effects or opioid use. In this case, CHS was not initially suspected; however, it became a leading diagnostic consideration as the patient’s vomiting persisted despite multiple antiemetics, and her chronic cannabis use emerged as a clinically significant factor during a detailed reevaluation.

Understanding the pathophysiology of CHS is critical for distinguishing it from routine postoperative complications. CHS is believed to result from overstimulation and eventual downregulation of CB1 receptors in the gastrointestinal tract and hypothalamus, leading to impaired gastric motility and dysregulation of the emetic reflex [7]. Functional MRI studies have supported this theory, showing that chronic tetrahydrocannabinol (THC) exposure alters both central and enteric cannabinoid signaling pathways [8]. This dysregulation is thought to underlie the paradoxical development of hyperemesis in habitual cannabis users, despite cannabis's known antiemetic properties.

One of the most important teaching points is the tendency for both patients and clinicians to dismiss cannabis as a contributing factor due to its well-known antiemetic properties. This misconception frequently delays diagnosis. In fact, a systematic review reported a median delay of 4.1 years from symptom onset to diagnosis of CHS, with many patients undergoing unnecessary and extensive diagnostic workups during that period [4]. In perioperative contexts, such delays can be especially costly, resulting in extended hospital stays, unwarranted imaging, and inappropriate medication adjustments that fail to address the underlying etiology [2,4]. Beyond its emetogenic potential, chronic cannabis use may also affect anesthetic susceptibility and opioid requirements, further complicating perioperative care [4].

A clinical pearl in this case was the patient’s report of compulsive hot showering, which she used for symptom relief prior to surgery. This behavior, a hallmark feature of CHS, is believed to reflect temporary modulation of thermoregulatory pathways in the hypothalamus by external heat stimuli [3,9]. Including this diagnostic clue early in the clinical course can help redirect diagnostic efforts and reduce unnecessary interventions. Importantly, while supportive care aids recovery, definitive treatment requires complete cessation of cannabis use. In this case, enforced abstinence during hospitalization and patient education led to rapid improvement, consistent with prior case reports [10].

With cannabis use becoming increasingly prevalent across age groups and geographic regions, it is imperative that perioperative teams maintain a high index of suspicion for CHS when evaluating refractory postoperative nausea and vomiting. Routine screening for chronic cannabis use should be integrated into preoperative assessments, involving multidisciplinary teams including anesthesia, surgery, and nursing. These professionals should be educated on the signs, symptoms, and clinical course of CHS. Early recognition can prevent unnecessary interventions, reduce healthcare costs, and significantly improve patient outcomes. Moreover, incorporating patient education and public health initiatives focused on cannabis-related risks can foster greater patient honesty regarding cannabis use and support preventive strategies, ultimately enhancing perioperative care [4].

## Conclusions

In cases of PONV that are refractory to standard antiemetic therapy, CHS should be considered, particularly in patients with a history of chronic or daily cannabis use. Differentiating CHS from more common postoperative conditions, such as opioid-induced nausea, is essential. While opioid-related emesis often improves with dose reduction or a change in agent, CHS typically persists until cannabis use is discontinued. A detailed clinical history, including patterns of cannabis intake, use of compulsive hot-water bathing for symptom relief, and the timing of emesis in relation to opioid administration, can provide key diagnostic clues and support early recognition.

Heightened clinical awareness of CHS in perioperative care can reduce unnecessary diagnostic testing, avoid ineffective medication changes, and facilitate timely interventions. Most importantly, early identification of CHS allows clinicians to educate patients on the underlying cause of their symptoms and support sustained cannabis cessation. As cannabis use becomes increasingly common, particularly in surgical populations, routine screening and clinician education will be critical tools in improving perioperative outcomes.

## References

[1] Allen JH, de Moore GM, Heddle R, Twartz JC (2004). Cannabinoid hyperemesis: cyclical hyperemesis in association with chronic cannabis abuse. Gut.

[2] Richards JR (2018). Cannabinoid hyperemesis syndrome: pathophysiology and treatment in the emergency department. J Emerg Med.

[3] Simonetto DA, Oxentenko AS, Herman ML, Szostek JH (2012). Cannabinoid hyperemesis: a case series of 98 patients. Mayo Clin Proc.

[4] Sorensen CJ, DeSanto K, Borgelt L, Phillips KT, Monte AA (2017). Cannabinoid hyperemesis syndrome: diagnosis, pathophysiology, and treatment-a systematic review. J Med Toxicol.

[5] Stanghellini V, Chan FK, Hasler WL, Malagelada JR, Suzuki H, Tack J, Talley NJ (2016). Gastroduodenal disorders. Gastroenterology.

[6] Peles S, Khalife R, Magliocco A (2025). Cannabinoid hyperemesis syndrome: a rising complication. Cureus.

[7] Hejazi RA, Lavenbarg TH, McCallum RW (2010). Spectrum of gastric emptying patterns in adult patients with cyclic vomiting syndrome. Neurogastroenterol Motil.

[8] Venkatesan T, Levinthal DJ, Li BU (2019). Role of chronic cannabis use: cyclic vomiting syndrome vs cannabinoid hyperemesis syndrome. Neurogastroenterol Motil.

[9] Namin F, Patel J, Lin Z, Sarosiek I, Foran P, Esmaeili P, McCallum R (2007). Clinical, psychiatric and manometric profile of cyclic vomiting syndrome in adults and response to tricyclic therapy. Neurogastroenterol Motil.

[10] Moon AM, Buckley SA, Mark NM (2018). Successful treatment of cannabinoid hyperemesis syndrome with topical capsaicin. ACG Case Rep J.

